# A Systematic Review of Research on Family Resemblance Approach to Nature of Science in Science Education

**DOI:** 10.1007/s11191-022-00379-3

**Published:** 2022-08-25

**Authors:** Kason Ka Ching Cheung, Sibel Erduran

**Affiliations:** Department of Education, University of Oxford, 15 Norham Gardens, Oxford, OX2 6PY UK

**Keywords:** Nature of science, Family resemblance approach, Cognitive-epistemic system, Social-institutional system, Systematic literature review

## Abstract

The paper reports about the outcome of a systematic review of research on family resemblance approach (FRA) to nature of science in (NOS) science education. FRA is a relatively recent perspective on NOS being a system of cognitive-epistemic and social-institutional aspects of science. FRA thus consists of a set of categories such as aims and values, practices, knowledge and social organizations in relation to NOS. Since the introduction of the FRA, there has been increasing interest in investigations about how FRA can be of use in science education both empirically and practically. A journal content analysis was conducted in order to investigate which FRA categories are covered in journal articles and to identify the characteristics of the studies that have used FRA. These characteristics included the target level of education and focus on pre- or in-service teachers. Furthermore, epistemic network analysis of theoretical and empirical papers was conducted to determine the extent to which the studies incorporated various key themes about FRA, such as its transferability to other domains and differentiation of the social-institutional system categories. The findings illustrate an increasing number of empirical studies using FRA in recent years and broad coverage in science education. Although the social-institutional system categories included intraconnections, these were not as strong as those intraconnections among categories within the cognitive-epistemic system. Future research directions for the use of FRA in K-12 science education are discussed.

## Introduction

Since the introduction of the family resemblance approach (FRA) to nature of science (NOS) in science education (Irzik & Nola, 2011, 2014; Erduran & Dagher, 2014), there has been increasing interest in investigating how FRA can be of use in science education both empirically and practically. FRA posits that different science disciplines having their own characteristics as well as general features. In Irzik & Nola’s (2011) characterization of FRA, similarities and differences across science discipline are captured across a range of aspects such as scientific knowledge, ethos and values. The family resemblance idea was inspired by Wittgenstein (1958) work. Just as members of a biological family can share some similar characteristics with one another, such as eye colour and shape of the nose, other characteristics will also differ from each other, for example the distinct facial features. In order to illustrate the family resemblance idea, Irzik and Nola, (2011) explained an example of games from Wittgenstein. The term “game” can include ball games, stick games and solo games which share some but not all characteristics. Similarly, for the term “science” or “nature of science”, different disciplines have different scientific activities.

In astronomy, astronomists use telescopes to study the space; in molecular biology, biologists investigate DNA through gel electrophoresis; and in chemistry, chemists use gas chromatography to identify substances. Experimenting can be a common characteristic among chemistry and biology, but not in astronomy except perhaps in modelling contexts. However, experimenting can still be one of the categories of NOS since it is not necessary for one characteristic of science to be generalized across all disciplines of science. In short, family resemblance approach provides a set of categories that reflect the domain-specific and domain-general characteristics of science.

Inspired by Irzik and Nola’s (2011) original account of FRA, Erduran and Dagher (2014) significantly extended the framework and infused it with research literature in science education to illustrate the relevance and potential of FRA for science education. Erduran and Dagher’s (2014) book-length account of FRA characterizes NOS as a cognitive-epistemic and a social-institutional system. The first set of categories related to *cognitive-epistemic system* consists of four categories: aims and values, methods and methodological rules, scientific knowledge and scientific practices. The second set of categories refers to *social-institutional system*, which consists of another seven categories: professional activities, scientific ethos, social certification and dissemination of scientific knowledge, social values, social organizations and interactions, political power structures and financial systems. Each FRA category is unpacked extensively to illustrate how they are justified through research in philosophy of science as well as science education, making links to the theoretical underpinnings of FRA as well as its empirical relevance in science education research traditions. For example, the social-institutional system categories are related to research in socio-scientific issues (Zeidler et al., 2005) and activism (Bencze et al., 2012) as well as science-technology-society studies (Aikenhead, 1994).

A broad review on the applications of FRA in science education is available (Erduran et al., 2019) although this review was published about 5 years ago and it is conceivable that more studies have been carried out about FRA in science education in the meantime. Furthermore, as far as we are aware, there are no studies in the literature that have adopted a systematic approach to review theoretical and empirical research based on FRA in science education. A systematic review will illustrate the research trends which can point to potential new areas that may have been under-investigated. The primary objective of this paper, then, is to develop, apply and report about a methodological approach to systematic literature review on the coverage of FRA in science education research in order to trace the trends in how the literature has engaged with the FRA since its introduction in science education.

In this paper, we use epistemic network analysis as a tool to compare the connections of advantages of using FRA in both theoretical and empirical studies. Following a comparison between theoretical and empirical studies, some suggestions on which FRA categories and how these categories can be addressed in these two different types of studies will be proposed. The following three interrelated research questions guided the systematic review:

1. Which FRA categories are covered in the reviewed studies?
2. What are the characteristics of studies that have utilized FRA as a NOS framework?
3. What are the strengths of FRA addressed in theoretical and methodological studies?

## Research on FRA in Science Education

Since Irzik and Nola’s (2011) original proposal about FRA as well as their subsequent extension of the framework (Irzik & Nola, 2014) and the book length account by Erduran and Dagher (2014), researchers have utilized FRA in different educational contexts. FRA has been adopted as a framework to investigate the content of science curricula in different parts of the world including Ireland (Erduran & Dagher, 2014), Turkey (Kaya & Erduran, 2016), Taiwan (Yeh et al., 2019), Italy (Caramaschi et al., 2022) and South Korea (Park et al., 2020a, 2020b). Further studies have included analysis of textbooks (e.g. BouJaoude et al., 2017; McDonald, 2017) as well as the development of practical resources including instructional materials (Erduran et al., 2019) and professional development resources Erduran and Kaya, 2018) . Erduran et al. (2021a) as well as Dagher (2020) investigated how the FRA framework could be linked to broader curricular goals related to social justice. University students’ understanding of NOS has been investigated using the FRA framework (Akgun & Kaya, 2020) and the utility of FRA in the enculturation of university students in scientific cultures has been explored (Mohan & Kelly, 2020).

Numerous analytical tools have been generated capitalizing on FRA. Quantitative tools have included a questionnaire that investigated pre-service teachers’ understanding of NOS based on the cognitive-epistemic and social-institutional dimensions (Kaya et al., 2018) as well as those that focused on particular FRA categories such as the social-institutional systems (Akbayrak & Kaya, 2020), aims and values (Kelly & Erduran, 2019), financial systems (Kaya et al., 2018) and scientific methods (El Masri et al., 2021; Erduran et al., 2021a, 2021b; Ioannidou & Erduran, 2020). Qualitative methods have included the use of interviews (Erduran et al., 2019) as well as the FRA wheel (Erduran & Dagher, 2014) as a mean to elicit group discussions (Erduran et al., 2021a, 2021b). Drawings of FRA categories by pre-service teachers have been to elicit their emerging and changing understandings of NOS (Erduran & Kaya, 2018). Mixed methods approaches have included the applications of epistemic network analysis on FRA categories (Cheung, 2020). Document analysis based on analytical categories derived from FRA has been used to trace the content of science curricula (Yeh et al., 2019) and textbooks (Park et al., 2020a, 2020b).

The FRA has the potential to be extended for improving understanding and for conceptual clarification outside the domain of science. The categories in the FRA are not solely descriptive in nature, but they represent a class of ideas (Erduran et al., 2019) which can be extended to other domains. Erduran et al. (2020) showed that the categories in the FRA to NOS can also be mapped to social justice concepts. Puttick and Cullinane (2022) argued that the categories in the FRA to NOS could also be applied to geography education. Some researchers who are interested in the FRA have a strong commitment to extend NOS understanding to functional understanding. For example, drawing on Bloom’s taxonomy, Cheung (2020) examines how different skills of FRA understanding can be assessed in summative assessments. Park et al., (2020a, 2020b) analysed how Korean textbook tasks elicited students’ application and evaluation of NOS based on the framework of the FRA.

The FRA to NOS is supported by a wealth of empirical evidence, robust theoretical rationale by philosophers of science and affirmation by practicing scientists themselves (e.g. Wu & Erduran, 2022). Although the FRA to NOS has received a few criticisms, these criticisms have been limited owing to their small number of published papers and their underdeveloped discussion of the FRA to NOS. For example, some authors (e.g. McComas, 2020) have suggested that FRA offers nothing new to science education research on NOS without a substantial account to provide a rationale for such a claim. Others (e.g. Kampourakis, 2016) have claimed that FRA can be taught at more advanced stages of education, suggesting that it is too complex for school science. Such claims have not been based on any empirical evidence and remain to be investigated, for example through research studies that compare the uptake of different models of NOS in students’ learning outcomes through classroom-based research.

In one paper, do Nascimento Rocha and Gurgel (2017) offered two criticisms on both FRA and consensus views. In their account, both consensus view and the FRA lead to students learning a particular meaning of science, and help students internalize NOS understanding at descriptive level *only* (do Nascimento Rocha & Gurgel, 2017). However, this criticism neglects the fact that the FRA to NOS could be a *theoretical* and *methodological* approach to curriculum, assessment, teacher education and classroom intervention of the epistemology of science (Erduran et al., 2019). As a theoretical and methodological approach, the FRA does not intend to prescribe a static and non-interactive feature that defines NOS. FRA has been adopted to identify the *fluid* and *dynamic* epistemic underpinnings across different STEM disciplines (Park et al., 2020a, 2020b).

Despite some criticisms, the FRA inherits multiple strengths and can be applied to various domains and contexts of education. However, as far as we are aware, there is not any systematic review which documents the studies of this line of research. Content analysis of academic journals is an important aspect of educational research (Chang et al., 2010) and review articles are often represented in science education journals including *Science & Education* (e.g. Wang et al., 2022). Systematic review of journal articles provides researchers with insight into recent and emerging trends (Lin et al., 2014). Foreman-Peck and Winch (2010) note that content analysis of journals can provide evidence-based indicators for not only the status quo of research but also where future research can potentially be directed to impact educational practice.

The systematic review presented in this paper has three overarching purposes. Firstly, it explores which categories of the FRA to NOS were addressed in these articles. Secondly, it reveals the major features (Chang et al., 2010) of empirical studies on the FRA to NOS, including the type of the studies, their participants, the domain of science addressed, artefacts collected and how these studies analyse and present the results. Thirdly, in responses to the criticism, we illustrate how researchers capitalize six major strengths of the FRA to NOS in their studies: (a) delimits descriptive understanding and extends understanding of NOS to other types understanding; (b) supports visualization of NOS categories (Erduran & Dagher, 2014); (c) captures domain-specific and domain-general nature of science (Park et al., 2020a, 2020b); (d) fosters transferability of the FRA to other disciplines; (e) demonstrates connections among NOS; and (f) differentiates finer categories in the social-institutional system.

## Methodology

### Systematic Review Strategy

**Fig. 1:**
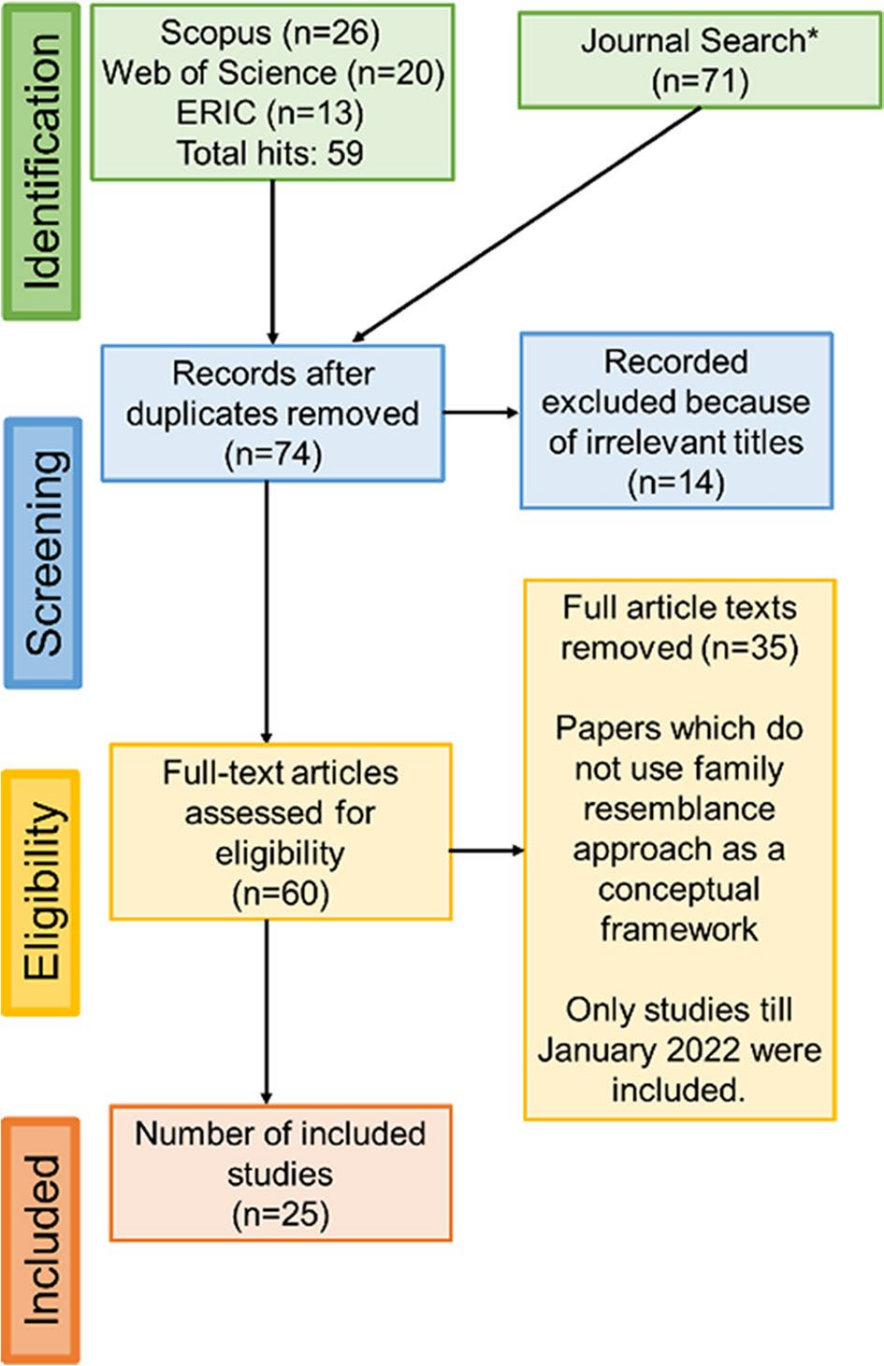
A four-stage flow diagram of systematic review. The figure shows stages of identifying studies, screening studies, assessing their eligibility and the final number of studies included

The selection criteria and procedures for identifying literature followed a systematic review protocol, namely the Preferred Reporting Items for Systematic Reviews and Meta-Analyses (PRISMA) statement (Moher et al., 2009). The PRISMA statement comprises a four-stage flow diagram of systematic review: identification, screening, searching for eligibility, inclusion of articles of interest (see Fig. 1). The PRISMA statement also comprises 27 checklist items. Although the PRISMA statement is not a checklist for assessing the quality of systematic review, it helps authors improve their practice of reporting systematic review. The major aim of our search was to identify studies that use the FRA to NOS as their conceptual framing. Both empirical and theoretical studies were traced. Book chapters, editorials and book reviews were excluded. It should be noted that there are books (e.g. Erduran & Dagher, 2014) as well as book chapters (e.g. Couso & Simmaro, 2020; Dagher, 2020; Erduran et al., 2020) that have been used FRA in science education research which are not captured in our review. In order to answer the research questions, we input the pair of keywords “nature of science” and “family resemblance approach” in searching Scopus, Web of Science and ERIC. We chose these databases because they typically index high-quality articles. The start year of publication was not restricted in the search because we wanted to capture as many articles as possible given the recent emergence of FRA in science education. The search was carried out in January 2022 so papers that may have been published following this date are not included. We are aware that at the time of the revision of the paper for publication, further studies have now been published not only in English language journals (e.g. Çilekrenkli & Kaya, 2022; Mork et al., 2022; Reinisch & Fricke, 2022) but also others (e.g. Shun-Qin et al., 2022). Hence, our review is likely to omit more recent publications that have been produced following our analysis in the lead up to the submission of this paper.

In our preliminary search, a total of 59 hits were indexed in three databases as follows: Scopus (*n* = 26), Web of Science (*n* = 20) and ERIC (*n* = 13). Given the small number of articles, we decided to extend our search specifically to journals that are related to science education.

The research team consisted of the authors of the paper who are two academics working in the field of NOS. We proposed a list of journals and discussed the possibility of whether these journals included studies on FRA. Considering the representation of some prominent journals in other researchers’ work (e.g. Lee et al., 2009) as well as conference programs in major science education research associations such as NARST and ESERA, we classified the journals into four categories: (1) science education journals (*Journal of Research in Science Teaching*, *Science Education*, *Research in Science Education*, *International Journal of Science Education*, *Science and Education* and *Journal of Science Teacher Education*); (2) interdisciplinary science education journals (*International Journal of Science and Mathematics Education*, *Research in Science and Technological Education*, *International Journal of STEM Education*); (3) discipline-specific science education journals (*Journal of Biological Education*, *Chemistry Education Research and Practice*, *Physics Review Education Research*); and (4) educational philosophy journals (*Educational Philosophy and Theory* and *Interchange*). The primary inclusion criterion of articles was whether or not they were indexed in SSCI, Web of Science or Scopus. After inputting the same set of keywords (“nature of science” and “family resemblance approach”), the results yield a total of 25 articles when duplicates were removed, and the eligibility of the manuscripts was assessed. It should be noted that some educational research journals have not been included as we wanted to focus our attention to science education outlets. This focus may have excluded some articles that have focused on FRA in science education but were published in generic education journals (e.g. Kaya-Capocci et al., 2021; Kelly & Erduran, 2019).

### Development of the Analysis Schemes

We searched different literature databases and journal databases that focused on the FRA to NOS. The following sections elaborate on how we carried out the search including the inclusion and exclusion criteria as well as the procedure of developing a coding framework. The formulation of the coding scheme was guided by the three research questions. Table 1 shows the coding scheme for analysing the selected articles.

**Table 1:** Analysing empirical studies

Research questions	Categories	Codes
1. Which FRA categories are covered in the reviewed studies?	(1) Cognitive-epistemic system	(a) Aims and values (b) Methods and methodological rules (c) Practices (d) Knowledge
	(2) Social-institutional system	(a) Social certification and dissemination (b) Professional activities (c) Scientific ethos (d) Social values (e) Political power structures (f) Financial systems (g) Social organizations and interactions
2. What are the characteristics of studies that have utilized FRA as a NOS framework?	(1) Type of studies	(a) Theoretical papers (b) Curriculum materials analysis (c) Educational intervention (d) Students’ and teachers’ understanding of nature of science (e) Instrument development (f) Informal science education
	(2) Participants	(a) Elementary students (b) Primary students (c) Secondary students (d) University students (e) Pre-service teachers (f) In-service teachers (g) General public
	(3) Domain of science	(a) Chemistry (b) Biology (c) Physics (d) Engineering (e) Mathematics (f) Technology (g) General Science (h) Others (please specify: ____________)
	(4) Artefacts collected	(a) Interviews (b) Questionnaires (i) Open-ended questionnaire (ii) Likert-scale questionnaire (c) Multimodal representations (d) Documents (i.e. curriculum/assignments) (e) Others (please specify: _____________________)
	(5) Analysis and presentation of results	(a) Graphs (b) Frequency of codes (c) Descriptive Statistics (d) Multivariate statistics (e) Epistemic networks (f) Others (please specify: _____________________)
3. What are the strengths of FRA addressed in theoretical and methodological studies?		(a) Delimits descriptive understanding and extends understanding of NOS to other types understanding (b) Supports visualization of NOS categories (Erduran & Dagher, 2014) (c) Captures domain-specific and domain-general nature of science (Park et al., 2020a, 2020b) (d) Fosters transferability of the FRA to other disciplines (e) Demonstrates connections among NOS Connections among NOS categories (f) Differentiates finer categories in the social-institutional system
		(Please write here)

The coding scheme for the first research question was produced deductively in relation to the FRA framework itself (Erduran & Dagher, 2014), while the codes for the second and third research questions were generated inductively by constant comparison of the content of surveyed studies (Braun & Clarke, 2006). In order to answer the first research question, the codes included all FRA categories (Erduran & Dagher, 2014). In order to address the second research question, 5 categories were generated: (1) type of studies; (2) participants; (3) domain of science; (4) artefacts collected; and (5) analysis and presentation of results. If the paper was theoretical, items (2) to (5) were not applied as they did not involve primary data collection. The third research question was addressed by analysing items (1) and (2). The coding scheme also took into consideration a broad range of other aspects that are pertinent to science education in general, including the level of education (i.e. secondary schooling, tertiary education) and career stages of teachers (i.e. pre-service, in-service teachers). The methodological approaches of the studies including their use of qualitative and quantitative methods were also captured.

After developing the coding scheme, we discussed whether or not the codes captured the features of the studies using the FRA to NOS. In order to ensure reliability of the coding scheme, we discussed changes that should be made to the coding scheme following the review of several studies (Cheung & Tai, 2021). The original version of the coding scheme grouped pre-service teachers and in-service teachers as a single group. However, pre-service teachers and in-service teachers have different levels of understanding and pedagogical content knowledge of NOS (Cheung, 2018). Hence, we considered that it might be valuable to distinguish these two groups of teachers. Furthermore, we came across different studies on the nature of science, technology, engineering and mathematics. Therefore, apart from traditional domains of science, we created codes including engineering, mathematics and technology to capture any studies that are more broadly related to STEM and STEM education as well.

### Epistemic Network Analysis

A significant aspect of Erduran and Dagher’s (2014) characterization of FRA is the interconnections between the FRA categories. In other words, these authors argue that for educational purposes, students’ meaning making about NOS would be enhanced if, for instance, they could understand what they are doing (e.g. scientific practices) in relation to the goals of investigations (e.g. aims and values) situated in relevant contexts (e.g. social organizations and interactions). Hence, one aspect of the first research question was the extent to which not only the FRA categories are represented in the articles but also how these papers may have made an effort to interrelate the FRA categories. In order to pursue this line of questioning, we have performed epistemic network analysis (ENA) on the identified articles. ENA has been used in science education research to establish connections between different concepts (e.g. Peters-Burton & Baynard, 2013; Cheung & Winterbottom, 2021) and it has been used in previous studies on FRA (e.g. Caramaschi et al., 2022; Author, 2020; Peters-Burton et al., 2022). ENA visualizes the *co-occurrences* between two codes in a stanza (Shaffer, 2017), which was defined as one single study identified in the systematic review procedure.

In epistemic networks, a thicker line indicates a stronger connection between two nodes, while a thinner line indicates a weaker connection between two nodes (Shaffer et al., 2016). This technique projects the connections of these codes in a high-dimensional space (Pantić et al., 2022). Two types of networks were created, a network showing how two NOS categories were simultaneously addressed in empirical studies (Caramaschi et al., 2022; Cheung, 2020; Gandolfi, 2021). Two networks created show how strengths of FRA were concurrently addressed in theoretical and empirical studies respectively. The former type of network shows how an empirical study investigates two concurrent NOS categories in FRA while the later type of framework enables comparison of how advantages of FRA were simultaneously capitalized in theoretical and empirical studies respectively. The thickness of connections, as well as network comparison, enables interpretation of meaning of patterns across studies.

## Results and Findings

**Fig. 2:**
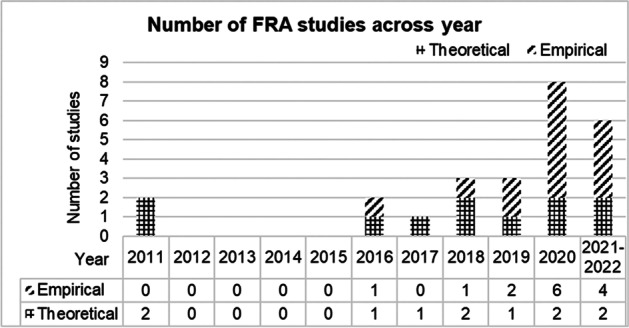
Distribution of FRA studies in science education from 2011 to January 2022

The analysis of the review data illustrates several trends. First, the number of studies using FRA in science education has increased in the last decade. This observation is particularly noteworthy in relation to empirical studies. Figure 2 highlights that between 2012 and 2015, there were no journal articles dedicated to FRA. It should be noted that the 2011 paper was Irzik and Nola’s (2011) seminal article which was subsequently revisited in a handbook chapter (Irzik & Nola, 2014) not captured in the data. Erduran and Dagher’s (2014) book is also not represented in the data since the search is exclusively dedicated to peer-reviewed research articles.

In addressing the first research question, Table 2 demonstrates that for the most part, the empirical studies capitalized on most FRA categories. Among the 14 empirical studies, four studies were found to address exclusively on the cognitive-epistemic categories of FRA; one study places an exclusive emphasis on the social-institutional categories of FRA; five studies addressed all categories of FRA; and four studies addressed some but not all categories in the cognitive-epistemic system and the social-institutional system. There are advantages in either addressing a few categories of FRA or all categories of FRA. In the work of Park et al., (2020a, 2020b), despite their analysis of two categories, they compared and contrasted in details how three STEM domains, science, mathematics and technology/engineering, are similar and different from each other in terms of aims and values and practices. An exclusive focus allows the authors to compare salient differences in curriculum documents in three nations. In contrast, Wu and Erduran (2022) compared scientists’ views of all FRA categories where scientists specialized in different disciplines. Such analysis facilitates illustration of how the cognitive-epistemic and the social-institutional aspects of NOS are represented in scientists’ understanding.

**Table 2:** Categories in the FRA addressed by empirical studies (*n* = 14)

Studies	Cognitive-epistemic system				Social-institutional system						
	Aims and values	Methods and methodological rules	Practices	Knowledge	Social certification and dissemination structures	Professional activities	Scientific ethos	Social values	Political power	Financial systems	Social organizations and interactions
Akbayrak and Kaya (2020)					X	X	X	X	X	X	X
Akgun and Kaya (2020)	X	X	X	X	X	X		X	X	X	X
Azninda and Sunarti (2021)	X	X	X	X							
Bichara et al. (2022)	X	X	X	X	X	X	X	X	X	X	X
Caramaschi et al. (2022)	X	X	X	X	X	X	X	X	X	X	X
Cheung (2020)	X	X	X	X	X	X	X	X	X	X	X
Erduran and Kaya (2018)			X	X							
Kaya et al. (2019)	X	X	X	X							
Kaya and Erduran (2016)	X	X	X	X	X	X	X	X	X	X	X
Park et al., (2020a, 2020b)	X		X								
Park et al., (2020a, 2020b)	X	X	X	X							X
Petersen et al. (2020)		X		X		X	X	X	X	X	X
Wu and Erduran (2022)	X	X	X	X	X	X	X	X	X	X	X
Yeh et al. (2019)	X	X	X	X	X		X	X	X		

Table 3 shows the results in relation to the second research question. There are examples of studies focusing on educational interventions (*n* = 4), curriculum analyses (*n* = 6), explorations of students and teachers’ understanding of NOS (*n* = 2), experts’ understanding of nature of science (*n* = 1) and informal science education (*n* = 1). In these studies, informal science education and experts’ understanding of NOS can be a potential research gap for researchers who are interested in FRA. Regarding informal science education, Bichara et al. (2022) examined how Covid-19 tweets reflected public engagement about NOS. Their study shows that FRA can be a theoretical tool for public science communication. Moreover, Wu and Erduran (2022) explored the utility of scientists’ view on FRA.

**Table 3:** The characteristics of empirical studies (*n* = 14) that adopt FRA as a conceptual framework

Studies	Type of studies	Participants	Domain of science/outside science	Artefacts collected	Analysis and presentation of results
Akbayrak and Kaya (2020)	Educational intervention	Secondary school students	Earth science	Interviews Likert-scale questionnaire	Graphs Descriptive statistics Multivariate statistics
Akgun and Kaya (2020)	Students’ and teachers’ understanding of nature of science	University students	General science	Interviews Likert-scale questionnaire	Frequency of codes Descriptive statistics
Azninda and Sunarti (2021)	Students’ and teachers’ understanding of nature of science	In-service teachers	General science	Interviews Likert-scale questionnaire	Graphs Frequency of codes Descriptive statistics
Bichara et al. (2022)	Informal science education	General public	General science	Tweets	Graphs Frequency of codes Descriptive statistics
Caramaschi et al. (2022)	Curriculum materials analysis	N/A	Physics	Documents	Frequency of codes Epistemic network analysis
Cheung (2020)	Curriculum materials analysis	N/A	Biology	Documents	Graphs Frequency of codes Epistemic network analysis
Erduran and Kaya (2018)	Educational intervention	Pre-service teachers	General science	Drawings Interviews	Tables comparing pre-post drawings
Kaya and Erduran (2016)	Curriculum materials analysis	N/A	General science	Documents	Table comparing different frameworks
Kaya et al. (2019)	Educational intervention	Pre-service teachers	General science	Interviews Likert-scale questionnaire	Descriptive statistics
Park et al., (2020a, 2020b)	Curriculum materials analysis	N/A	Engineering Mathematics Technology General science	Documents	Venn diagrams
Park et al., (2020a, 2020b)	Curriculum materials analysis	N/A	General Science	Documents	Graphs Frequency of codes
Petersen et al. (2020)	Educational intervention	University students	Biology	Likert-scale questionnaire	Graphs Descriptive Statistics
Wu and Erduran (2022)	Experts’ understanding of nature of science	Scientists	General science	Interviews	Graphs Frequency of codes
Yeh et al. (2019)	Curriculum materials analysis	N/A	Technology General science	Documents	Graphs Frequency of codes

As illustrated by Table 3, the science domains were diverse involving not only general science (*n* = 10) but also particular domains such as earth science (*n* = 1), biology (*n* = 2) and physics (*n* = 1). The number of studies about domain-specific NOS is fewer than those about domain-general nature of science. Two recent studies also focused on the application of FRA in STEM disciplines (Park et al., 2020a, 2020b). This observation shows that FRA is not only applied to different science disciplines, but it can also be applied to disciplines related to technology, engineering and mathematics. From a methodological perspective, FRA studies have used a range of methods such as interviews (*n* = 6) and questionnaires (*n* = 5) as well as documentary sources (*n* = 6). One study used drawings to explore pre-service teachers’ understanding of FRA after an intervention (Erduran & Kaya, 2018). The papers utilized a range of analysis techniques including descriptive statistics (*n* = 6), multivariate statistics (*n* = 1) and drawings (*n* = 1). Other modes of artefacts such as drawings can afford analysis of students and teachers’ understanding of FRA.

Tables 4 and 5 illustrate how the different papers focused on various aspects of FRA in order to reinforce particular strengths of the framework. For example, as shown in Table 4, there have been empirical studies that focused on the connections between the FRA categories (*n* = 5) as well as the theme of domain-generality and domain-specificity of NOS in the science curriculum (*n* = 6). In Table 5, there have also been theoretical studies that focus on the transferability and applicability of FRA to other non-science disciplines (*n* = 6) as well as the domain-generality and domain-specificity of NOS in the science curriculum (*n* = 5). The domain-general and domain-specific nature of FRA is addressed in a significant number of theoretical and empirical studies.

**Table 4:** The ways of empirical studies reinforce on the strengths of the FRA approach

*Studies*	*Main strengths of the FRA*						*Explanation of how these empirical studies reinforce/add on the FRA approach*
	(a) Delimits descriptive understanding and extends understanding of NOS to other types understanding	(b) Visualization of NOS categories (Erduran & Dagher, 2014)	(c) Captures domain-specific and domain-general nature of science (Park et al., 2020a, 2020b)	(d) Fosters transferability of FRA to other disciplines	(e) Demonstrates connections among NOS categories	(f) Differentiates finer categories in the social-institutional system	
Akbayrak and Kaya (2020)						X	• Integrates finer social-institutional aspects of science will improve students’ understandings of social dimensions of science
Akgun and Kaya (2020)					X		• Argues that perceptions of undergraduates are linked to their domain-specific backgrounds • Students made connections among categories in the Benzene Ring Heuristics
Azninda and Sunarti (2021)						X	• Teachers cannot provide examples and explanations of the social-institutional aspects of NOS
Bichara et al. (2022)					X	X	• Explores discourse in Twitter from a FRA perspective and identifies interrelationships among NOS categories • Identifies many tweets that are closely associated with different categories within the social-institutional system
Caramaschi et al. (2022)			X		X		• Makes use the domain-general and domain-specific characteristics to analyse general and specific sections of physics curriculum • Combines the use of FRA and epistemic network analysis to investigate the interconnections among NOS categories
Cheung (2020)					X	X	• Compare connections among NOS categories between summative assessment and curriculum documents • Analyses the categories in social-institutional system in a fine-grained manner
Erduran and Kaya (2018)		X					• Illustrates how visualization can be applied to nature of science pre-service teacher education • Shows improvement in pre-service teachers’ visualization of nature of science after teaching intervention
Kaya and Erduran (2016)						X	• Uses the FRA to compare Turkish and Irish curriculum • Some categories in the social-institutional system were underemphasized in both curricula
Kaya et al. (2019)	X						• Utilizes the FRA in teacher education program in Turkey • The FRA strengthens teachers’ understandings of the pedagogies of NOS, the ways that NOS is included in the curriculum and knowledge of students learning NOS
Park et al., (2020a, 2020b)	X						• Explores several types of tasks that probe into students’ diverse understandings of NOS: guiding to NOS ideas, expanding NOS ideas, thinking critically about NOS ideas
Park et al., (2020a, 2020b)		X	X				• Illustrates that there is diversity in the way that epistemic aims, values and practices are discussed in these documents • Finds that curricula downplays the subtle nuances that distinguish the two disciplines and may give a misleading impression that science and engineering are essentially the same •Uses Venn diagrams to visualize the similarities and differences among STEM disciplines
Petersen et al. (2020)			X			X	• Designs teaching unit “basic genetics” based on domain-specific and domain-general approach • Designs teaching activities which are framed by the finer aspects of the social-institutional system
Wu and Erduran (2022)						X	• Compared the views of all FRA categories of scientists who specialize in different disciplines • Facilitates an analysis about how the cognitive-epistemic and the social-institutional aspects of NOS is represented in scientists’ understanding
Yeh et al. (2019)					X		• Identifies how connections among NOS categories are addressed in Taiwan curriculum documents

**Table 5:** The ways of theoretical studies (*n* = 11) reinforce on the strengths of the FRA approach

*Studies*	*Main strengths of the FRA*						*Explanation of how these theoretical studies reinforce/add on the FRA approach*
	(a) Delimits descriptive understanding and extends understanding of NOS to other types understanding	(b) Visualization of NOS categories (Erduran & Dagher, 2014)	(c) Captures domain-specific and domain-general nature of science (Park et al., 2020a, 2020b)	(d) Fosters transferability of FRA to other disciplines	(e) Demonstrates connections among NOS categories	(f) Differentiates finer categories in the social-institutional system	
Dagher and Erduran (2016)		X				X	• Justifies the affordances of FRA in curriculum and aligns it with USA reform curriculum documents • Argues the use of generative visual tools which support understandings of epistemic and social underpinnings of science
Dagher and Erduran (2017)				X			• Consolidate the strengths of FRA by incorporating the arguments from Hodson and Wong (2017) • Emphasize on systematicity of NOS which provides principal tools for decision-making in socio-scientific issues • The FRA provides a source of categories that helps differentiate science, pseudoscience and non-science
Erduran et al. (2019)			X				• Discusses examples of practical application of the FRA to NOS • Suggests future research direction of the FRA to NOS
Inêz et al. (2021)		X	X				• Capitalizes FRA as a visual tool and incorporates in the Integrative Model for Teaching NOS in Biological Education (IM-NOSBIO) • FRA accounts for both domain-general and domain-specific nature of biology in five areas: cells, evolution, genetics, organisms and ecology
Irzik and Nola (2011)			X		X		• Proposes four categories, “activities”, “aims and values”, “methodologies and methodological rules” and “products” • Argues that the FRA can identify similarities and differences across disciplines of science
Laherto et al. (2018)				X		X	• Aligns dimensions of Responsible Research and Innovation (RRI) to categories of the FRA to NOS • Conceptualizes the connections between RRI and the FRA to NOS in order to address RRI in classrooms
Ortiz-Revilla, Aduriz-Bravo, and Greca (2020)				X			• Applies the FRA to describe some features of the nature of STEM which informs the design of future of integrated STEM curriculum
Puttick and Cullinane (2022)				X			• Conceptualizes how the categories in the FRA to NOS can be applied to teaching nature of geography • Explores the possibility of inclusion of nature of geography in teaching resources, curriculum development and teacher education
Romero-Maltrana and Duarte (2020)			X	X			• Captures the shared similarities and differences between science and other non-human endeavours • Proposes “Aims”, “Methods”, “Activities” and “Products” as four “meta-categories” to distinguish similarities and differences among two science disciplines, or a science discipline and a non-science discipline
S. Kaya et al. (2018)				X		X	• Integrates entrepreneurship and economics of science into NOS by highlight the correspondence of each other • Proposes the SAMI framework to incorporate entrepreneurship, NOS and economics of science which makes contributions to the literature in science education
van Dijk (2011)			X				• The FRA is adequate for science education and public science communication

**Fig. 3:**
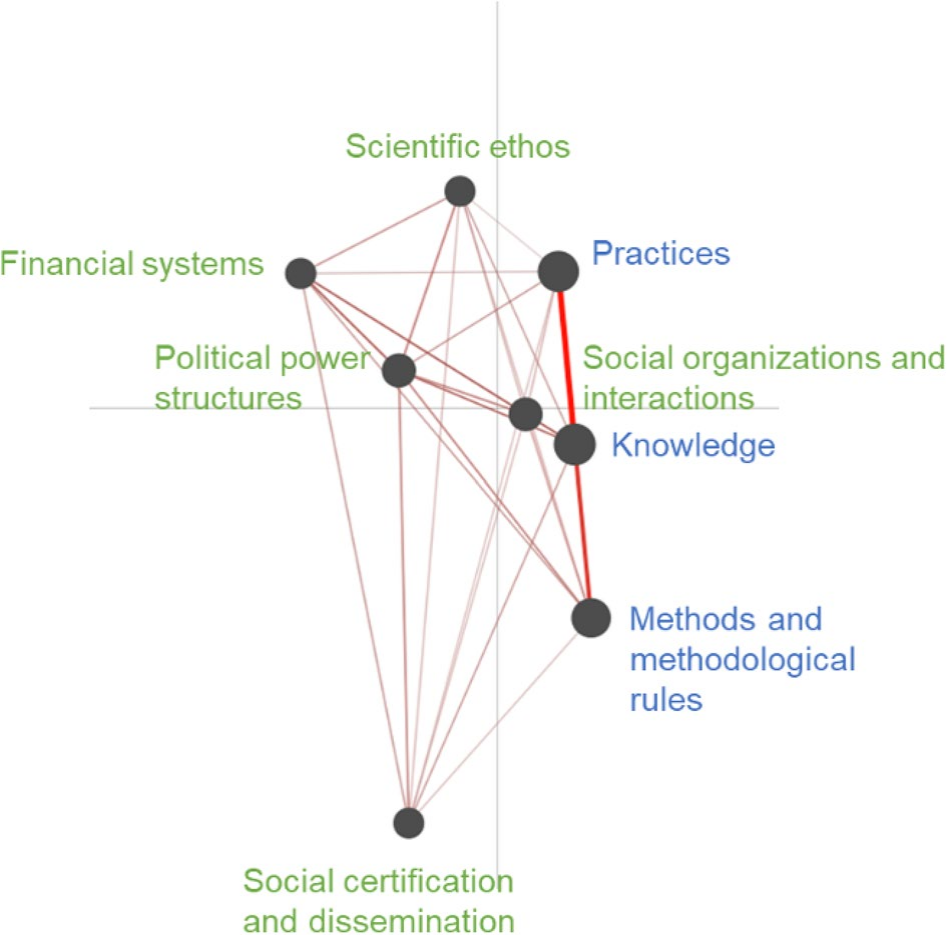
Epistemic networks showing how empirical studies (*n* = 14) simultaneously draw on categories of FRA. A thicker line indicates that there is a higher frequency for an empirical study to simultaneously examine two FRA categories. Nodes with blue texts indicate FRA categories within the cognitive-epistemic system, while nodes with green texts indicate FRA categories within the social-institutional system. In the diagram, there are comparatively strong connections between practices and knowledge, as well as knowledge and methods and methodological rules, as indicated by the thicker line

**Fig. 4:**
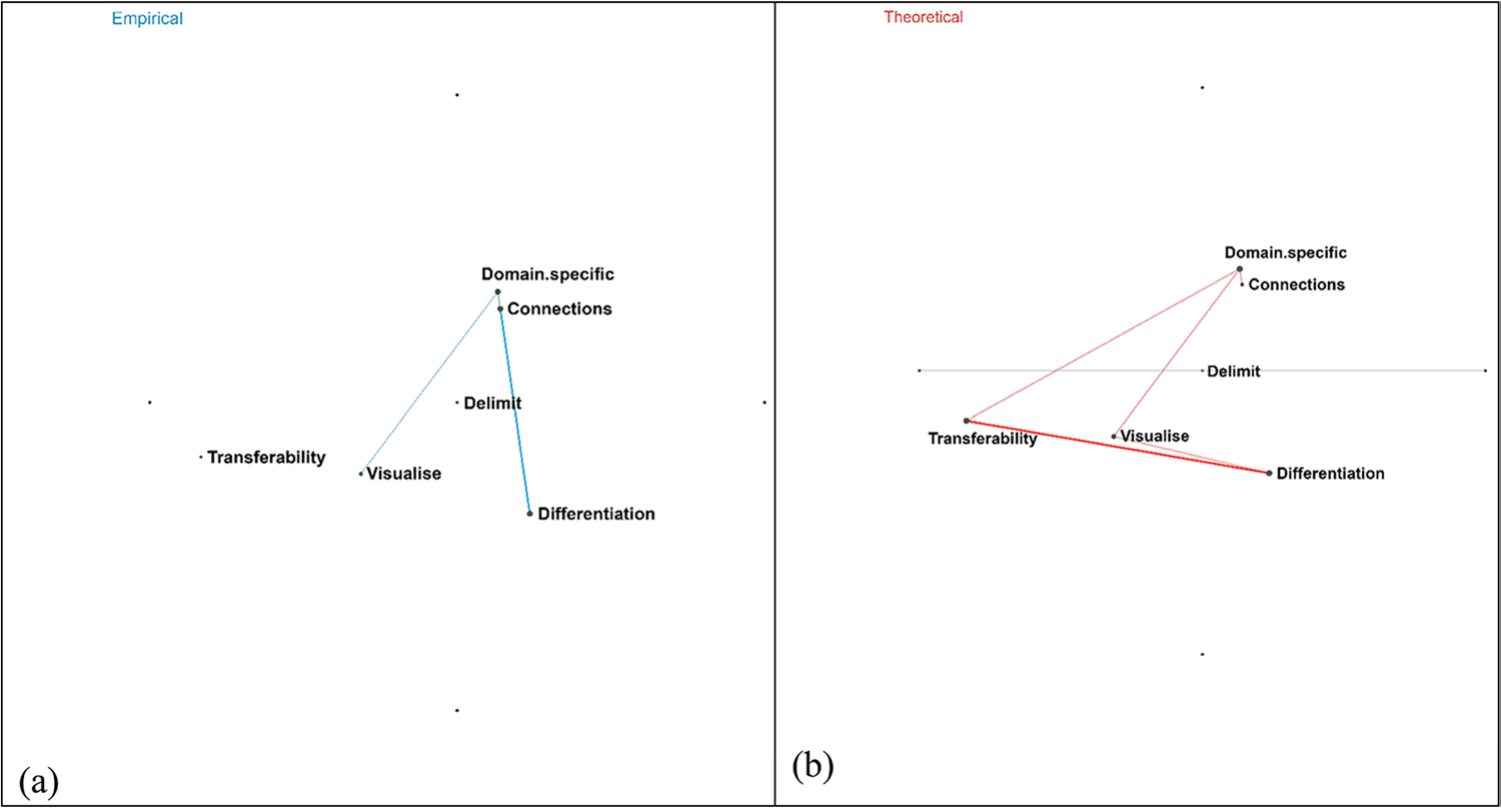
**A** Epistemic networks showing how empirical studies (*n* = 14) simultaneously draw on two advantages of FRA. A thicker line between “connections” and “differentiation” is observed, indicating a significant number of empirical studies simultaneously draw on the advantages of demonstrating connections among FRA categories and differentiating finer aspects of social-institutional categories. **b** Epistemic networks showing how theoretical studies (*n* = 11) simultaneously draw on two advantages of FRA. A thicker line between “transferability” and “differentiation” indicates that a significant number of theoretical studies simultaneously draw on the advantages of fostering transferability of FRA to other disciplines and differentiating finer categories in the social-institutional system

When the results of the ENA are reviewed, several trends can be observed. Figure 3 illustrates that stronger intraconnections are observed among categories in the cognitive-epistemic system, particularly with respect to the categories of practices, knowledge and methods and methodological rules. The intraconnections among categories in social-institutional system and interconnections of categories between two systems are also observed in these studies, but not as strong as the intraconnections among categories within the cognitive-epistemic system. Furthermore, Fig. 4a illustrates an epistemic network of how empirical studies (*n* = 14) simultaneously draw on two advantages of FRA, while Fig. 4b shows an epistemic network of how theoretical studies (*n* = 11) simultaneously drawing on two advantages of FRA. Theoretical studies frequently draw on both advantages of transferability and differentiation of social-institutional system of FRA while empirical studies draw on both advantages of connections among NOS categories and differentiation of social-institutional system of FRA.

## Conclusions and Discussion

Since the introduction of the FRA in science education research literature through the seminal paper by Irzik and Nola (2011) published in *Science & Education* in 2011, there have been a growing number of studies focusing on the adaptations of this framework in school-based research. The initial account was proposed by Irzik and Nola (2011, 2014) who are philosophers of science and subsequently the framework has been adapted and extended for science education in a book by Erduran and Dagher (2014). In this paper, we have investigated the coverage of FRA in science education journal articles through a systematic review of journals. It should be noted that the focus was on science education research journals so general education journals as well as book chapters were excluded in the systematic review, although we are aware that there is FRA-based research in such outlets as well (e.g. Dagher, 2020; Kaya-Capocci et al., 2021).

The findings illustrated that the empirical studies that have followed have focused on a range of issues such as education level and teachers’ career stages, and they have employed a range of methodological approaches including both qualitative and quantitative methodologies. An important finding is that there has been a range of curriculum analysis studies conducted using science curricula from different countries (e.g. Caramaschi et al., 2022; Park et al., 2020a, 2020b). These studies provide tangible recommendations for how science curricula could be reformed in order to make them more comprehensive and inclusive of different aspects of NOS. Interest in curriculum studies is continuing (e.g. Mork et al., 2022) which is not surprising given each national context presents particular nuances about what NOS is included in the curriculum, framing how science is taught and learned in a particular country. These observations point to the versatility of FRA for science education purposes. It is worthwhile to note that among the research studies, there has been a piece of research that has also investigated scientists’ own views of FRA. Wu and Erduran (2022) conducted an analysis of Taiwanese scientists and observed that scientists agree that the FRA account of NOS, and they detailed all aspects in their reference to NOS, although the social-institutional aspects were underrepresented in their depiction.

The results of the ENA highlighted stronger intraconnections among FRA categories in the cognitive-epistemic system, particularly with respect to practices, knowledge and methods and methodological rules. The intraconnections among categories in social-institutional system and interconnections of categories between two systems were also observed in these studies, but not as strong as the intraconnections among categories within the cognitive-epistemic system. This finding seems to be consistent with the underrepresentation of the social-institutional aspects of NOS in both the curriculum (Kaya & Erduran, 2016) and scientists’ accounts (Wu & Erduran, 2022) carried out with FRA as a guiding framework making these studies comparable in this sense. Further observations from ENA illustrated how both theoretical and empirical studies simultaneously draw on two advantages of FRA although they differ in terms of the nature of these advantages. While the theoretical studies frequently draw on both advantages of transferability and differentiation of social-institutional system of FRA, the empirical studies draw on both advantages of connections among NOS categories and differentiation of social-institutional system of FRA.

A potential affordance for these theoretical studies focusing on transferability is to explore the potential affordance of the conceptual framework of FRA to inform research agendas in other fields. For example, finer differentiation of social-institutional categories may facilitate the generation of new insights of in other fields of inquiry. For example, by using the FRA framework, Puttick and Cullinane (2022) explored how geography teaching can address the social-institutional aspects of geography. In discussing climate change, these researchers argued that explicit articulation of race and gender equality can potentially foster students’ understanding of political power structures, a construct that is part of the FRA framework proposed by Erduran and Dagher (2014). As such, adaptations of FRA in other domains from both a theoretical and empirical perspective may provide opportunities for novel research directions.

The methodology on the systematic review of the journal articles can potentially facilitate other researchers’ examination of trends in the literature particularly with respect to the identification of major connections between different themes that may be co-occurring with respect to aspects that are expected to be theoretically related, for instance in the case of how parts of a system relate to each other. Considering FRA is a relatively new addition to NOS studies in science education, it is expected that the synthesis provided in this paper will point to other studies in the future that will build on the applications of FRA in science education.

Based on the findings, four future research directions are suggested to maximize the potential contribution of FRA in science education. Firstly, researchers can extend empirical studies to settings in K-12 in order to determine the effectiveness of FRA-based interventions on student learning. Such studies are beginning to emerge in the literature (Çilekrenkli & Kaya, 2022) and further work is needed with respect to the explorations of the impact of FRA-informed instruction on students’ learning. Secondly, the design, implementation and testing of various models of integration of FRA can be pursued to identify how students’ understanding of different FRA categories can be enhanced. Thirdly, researchers can carry out fine-grained analysis of the social-institutional system categories in the learning environment. As exemplified by Table 2, only one study placed exclusive focus on social-institutional categories.

While previous related research such as socio-scientific issues (Zeidler et al., 2005) has capitalized on themes related to science and society, the FRA provides a delineation of the social context by offering a set of 7 different aspects that range from social values to social institutions. Such aspects of FRA may provide fruitful understanding of how to include under-represented features of science in science instruction. For example, although the Covid-19 pandemic has provided a fairly tangible account of the interactions of science with economics and politics, such aspects of NOS are still fairly underrepresented in science curricula across the world. The findings of the paper with regard to ENA point to the potential of this method in unison with FRA to illustrate certain patterns which can be traced in other areas of science education, for instance in investigating similarities and differences between teachers’ and students’ views of NOS.

Although some research is available in this area (e.g. Peters-Burton et al., 2022), the potential of FRA for tracing of students’ as well as teachers’ progression given particular interventions of learning has not yet been explored. Finally, one of the outcomes of our review was the identification of research that focused on a range of research instruments in identifying NOS understandings including, for example the use of drawings in tracing pre-service teachers’ drawings about NOS (Erduran et al., 2020). One study from Akbayrak and Kaya (2020) carried out educational intervention on K-12 students, and they measured their NOS understanding by questionnaires and interviews (Table 3). On the other hand, in the study by Erduran and Kaya (2018), pre-service teachers expressed their understanding through drawings. Therefore, future research programs can explore the use of different modes of representations through various instruments to investigate K-12 students’ and teachers’ understanding of NOS. Considering the limited emphasis on visual representations in relation to NOS, future research can develop further strategies and tools in order to explore the potential of visualization in relation to the teaching and learning of FRA categories in K-12 education.

While our analysis captures some publications on FRA in science education in recent years, as previously stated, we are aware that at the time of the revision of the paper for publication, further studies have now been published not only in English language journals (e.g. Çilekrenkli & Kaya, 2022; Mork et al., 2022; Reinisch & Fricke, 2022) but also others (e.g. Shun-Qin et al., 2022). Hence, our review omits more recent publications that have been produced following our analysis in the lead up to the submission and publication of this paper. Future studies can extend the timeframe of the systematic review to capture emerging research in the applications of FRA in science education.
